# Reconstruction of a midfacial defect using an intraoral-extraoral combination prosthesis employing magnets: a clinical report

**DOI:** 10.4317/jced.50715

**Published:** 2012-07-01

**Authors:** Anoop Nair, K M. Regish, Farhan K. Shah, D R. Prithviraj

**Affiliations:** 1 MDS, Senior Lecturer, Dept. of Prosthodontics. Govt. Dental College and Research Institute, Bangalore. Victoria Hospital Campus, Fort, Bangalore, India.; 2 BDS (MDS), Post Graduate, Dept. of Prosthodontics. Govt. Dental College and Research Institute, Bangalore. Victoria Hospital Campus, Fort, Bangalore, India.; 3 Senior Lecturer, Aligarh Dental College, Aligarh. India.; 4 Professor and Head. Dept. of Prosthodontics. Govt. Dental College and Research Institute, Bangalore. Victoria Hospital Campus, Fort, Bangalore, India.

## Abstract

Radical maxillectomy frequently leads to extended defects in hard and soft tissues that result in a connection between the oral cavities and orbit. If the defect cannot be surgically reconstructed, a combination prosthesis may be necessary to remedy dysfunction in patient function, comfort, esthetics. For minor defects, enlargement of the base of the intra oral prosthesis is generally sufficient. Resections that affect more than one third of the maxilla usually require an intra oral and an extra oral prosthesis that could be assembled and retained in the patient. This clinical report describes a technique of prosthetic rehabilitation of midfacial defect with a silicone orbital prosthesis and intra oral obturator that are retained by magnets.

** Key words:**Combination Prosthesis, Silicone Prosthesis, Magnets, obturators.

## Introduction

Head and neck cancer treatment frequently leaves the patient with some facial deformity due to extensive muscle and bone loss which, in turn, can cause the patient to become depressed and isolated (1). Midfacial defects form one such group of defects and they are facial defects that have an intraoral communication (2). Marunick et al (3) classified midfacial defects into 2 major categories: midline midfacial defects, which include the nose and/or upper lip; and lateral defects, which include the cheek and orbital contents. Combinations of these 2 categories also exist. Large defects that result from cancer treatment rarely are rehabilitated by surgical reconstruction alone; they usually require a facial prosthesis to restore function and appearance (2). Maxillofacial prostheses have the advantage of not only improving the patient’s appearance but also enabling early rehabilitation. These prostheses make it possible to inspect the affected area, shorten surgery and hospitalization time, lower treatment cost, and allow the patient to be psychosocially re-integrated more quickly. Fabrication of an extraoral facial prosthesis challenges the artistic ability of the prosthodontist (1). In addition to the extraoral prosthesis, an intraoral prosthesis such as an obturator is often needed to restore speech and swallowing in such patients (2). Retention of the prosthesis is also a difficult problem because of its size and weight. Securing it in place can be a formidable task (1). This clinical report describes a technique of prosthetic rehabilitation of midfacial defect with a silicone orbital prosthesis and intra oral obturator that are retained by magnets.

## Case Report

**Figure 1:**
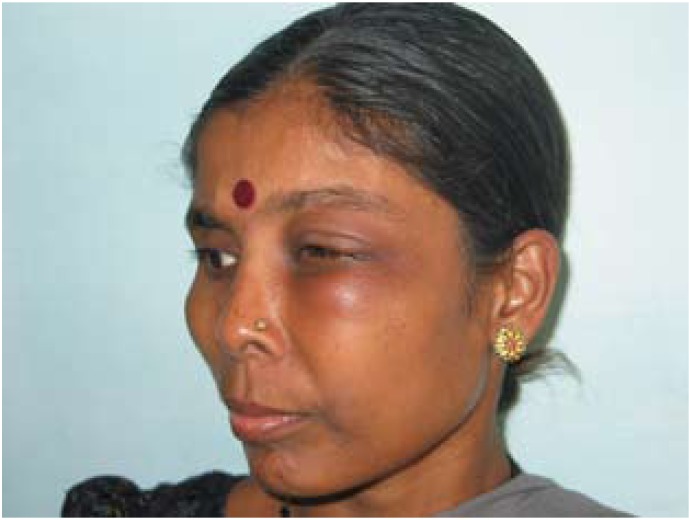
A 51 year old female patient.

A 51 year old female patient (Fig.1) was referred for definitive prosthetic rehabilitation after surgical excision of left maxilla including the orbit on the ipsilateral side along with its contents. Six Months after post operative healing a well healed surgical site with an oro-orbital defect requiring an orbital prosthesis and an intra oral prosthesis was seen. Decision for prosthetic rehabilitation was undertaken after ruling out surgical reconstruction owing to the financial constraints.

## Procedure

Intra oral impression was made with addition silicone (Aquasil, putty/lightbody) impression material and cast poured. The master cast was then duplicated to obtain a duplicate cast of defective site. A conventional record base with retentive terminals was fabricated on master cast and a Moloplast bulb separately in the duplicate cast. The Moloplast bulb was fabricated incorporating a circumferential retentive groove that can be used to mechanically retain it to the record base fabricated in the master cast. The record base, Moloplast assembly was then placed intraorally and a facial moulage made for the orbital prosthesis. The wax pattern for the orbital prosthesis was then fabricated on the cast extending throughout the defect and in contact with the moloplast bulb visible through the eye socket.

An acrylic shell was fabricated simulating sclera of the patient and an iris button of appropriate size was chosen and painted. During iris orientation, patient was asked to gaze straight ahead. The distance from the pupil of the normal eye to the midline was used in establishing the horizontal position of the prosthetic pupil’s centre. Its vertical position was determined by the canthus relationships. Marked coordinates of the pupil were used to circumscribe the diameter of the iris. Iris and scleral painting were carried out using acrylic colors and mo-no-poly. Subsequently the eye shell was packed with transparent acrylic to give a natural appearance. Later, excess was trimmed, finished and polished. The ocular prosthesis was then placed in the wax pattern and orien-ted using fixed guidelines.

**Figure 2:**
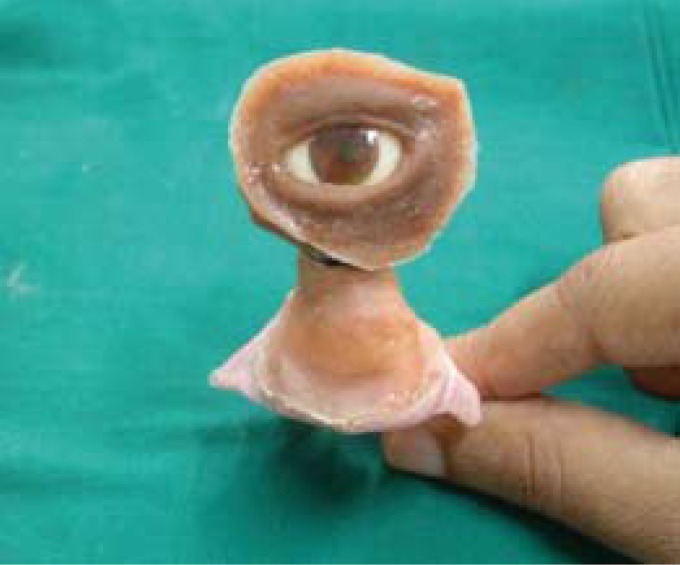
Silicone orbital prosthesis.

The entire wax pattern with the ocular prosthesis was lifted from the facial cast and invested and mould formed. Silicone material was then packed into the mould space to obtain a silicone orbital prosthesis. The prosthesis was then finished and polished (Fig. 2).

For retention mechanism, a Samarium Cobalt magnet was incorporated in the superior most portion of the moloplast bulb and the area of the silicone orbital prosthesis that is in close contact with the intra oral prosthesis.

Teeth were arranged in the intra oral prosthesis on the defective side using fixed guidelines to aid in the function comfort and esthetics in the patient.

**Figure 3:**
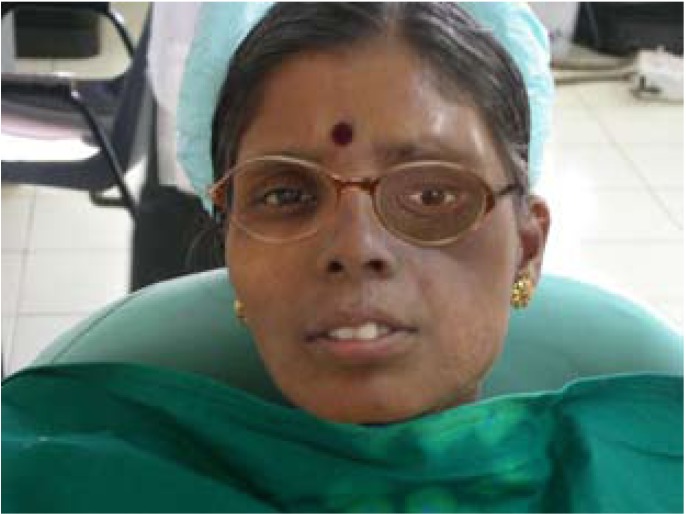
Spectacles to camouflage the scarred tissue.

The orbital prosthesis was then placed into the defect and denture placed intra orally, assembled using magnetic force and critically evaluated. Spectacles were used to camouflage the scarred tissue (Fig. 3).

## Discussion

Large orofacial defects can result in serious functional impairment of speech, mastication, and swallowing. The cosmetic deformity often has a significant psychological impact. Acceptable cosmetic results usually can be obtained, but retention of such a large prosthesis can be challenging. With ingenuity and an understanding of the remaining anatomic structures, intraoral and extraoral prostheses that mutually retain one another can be constructed with an appropriate choice of material and procedure (2). Silicones have been used for over 50 years in the field of maxillofacial prosthetics, with desirable material properties including flexibility, biocompatibility, ability to accept intrinsic and extrinsic colorants, chemical and physical inertness and mouldability (4,5). Heat cure polymethyl methacrylate was used for fabrication of the intra oral prosthesis which has better biocompatibility (6). Various methods of auxiliary retention for facial prostheses have been described in the literature; they include eyepatches, eyeglasses, extensions from the denture that engage tissue undercuts, magnets, adhesives, combinations of the above and osseointegrated implants. Although osseointegrated implants may provide the most reliable prosthesis retention, additional surgeries, expenses, inadequate bone, and prior radiation to the area may contraindicate this type of treatment (2). In such cases magnets (7) form the best alternative.

This case report describes the treatment of a patient with hemimaxillectomy and exenterated orbit using a prosthetic approach. Orbital Prosthesis was fabricated using medical grade silicone while an intraoral obturator was fabricated a self cure acrylic material and moloplast bulb. Both the prosthesis was oriented and retained using magnets. The prosthesis, although static, helped restore the patient’s appearance and confidence. In the absence of recurrent adenocarcinoma, this prosthesis can be a definitive treatment for the patient.

## Conclusion

Reconstruction of a large midfacial defect involving the orbit is a surgical challenge. Patients in such situation can be treated by giving an extra oral silicone orbital prosthesis and intra oral obturator prosthesis and retained using magnets.
